# Cholesterol Accelerates Aggregation of α-Synuclein Simultaneously Increasing the Toxicity of Amyloid Fibrils

**DOI:** 10.1021/acschemneuro.4c00501

**Published:** 2024-10-29

**Authors:** Mikhail Matveyenka, Abid Ali, Charles L. Mitchell, Harris C. Brown, Dmitry Kurouski

**Affiliations:** Department of Biochemistry and Biophysics, Texas A&M University, College Station, Texas 77843, United States

**Keywords:** α-synuclein, cholesterol, Caenorhabditis elegans, fibrils, ROS

## Abstract

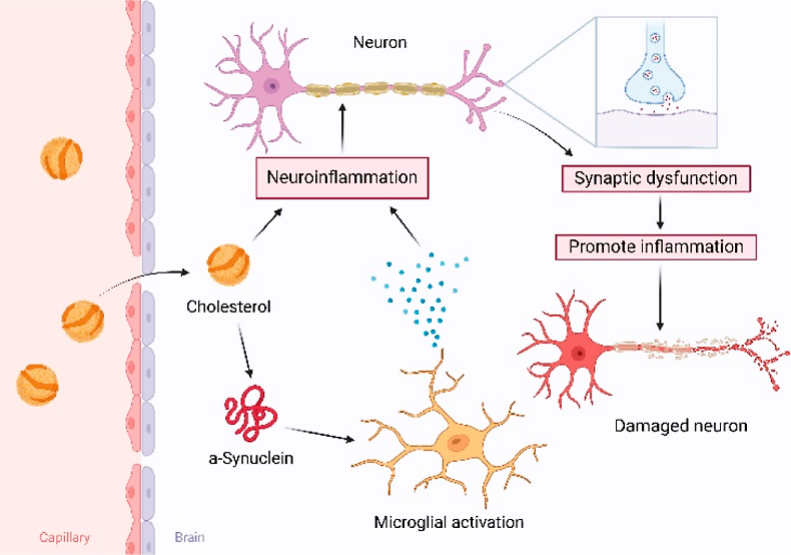

A hallmark of Parkinson disease (PD) is a progressive degeneration of neurons in the substantia nigra pars compacta, hypothalamus, and thalamus. Although the exact etiology of irreversible neuronal degeneration is unclear, a growing body of experimental evidence indicates that PD could be triggered by the abrupt aggregation of α-synuclein (α-Syn), a small membrane protein that is responsible for cell vesicle trafficking. Phospholipids uniquely alter the rate of α-Syn aggregation and, consequently, change the cytotoxicity of α-Syn oligomers and fibrils. However, the role of cholesterol in the aggregation of α-Syn remains unclear. In this study, we used *Caenorhabditis elegans* that overexpressed α-Syn to investigate the effect of low (15%), normal (30%), and high (60%) concentrations of cholesterol on α-Syn aggregation. We found that an increase in the concentration of cholesterol in diets substantially shortened the lifespan of *C. elegans*. Using biophysical methods, we also investigated the extent to which large unilamellar vesicles (LUVs) with low, normal, and high concentrations of cholesterol altered the rate of α-Syn aggregation. We found that only lipid membranes with a 60% concentration of cholesterol substantially accelerated the rate of protein aggregation. Cell assays revealed that α-Syn fibrils formed in the presence of LUVs with different concentrations of cholesterol exerted very similar levels of cytotoxicity to rat dopaminergic neurons. These results suggest that changes in the concentration of cholesterol in the plasma membrane, which in turn could be caused by nutritional preferences, could accelerate the onset and progression of PD.

## Introduction

Parkinson disease (PD), the fastest growing neurodegenerative disease, is projected to strike 12 million people by 2040 worldwide.^1^ In the U.S., 60,000 cases of PD are diagnosed annually with estimated costs that are upward of 30 billion, making effective neuroprotective treatments an urgent and unmet need.^2^ A hallmark of PD is accumulation of Lewy bodies in the midbrain, hypothalamus, and thalamus. These intracellular deposits are composed of lipid membranes and protein aggregates.^3^ This evidence suggests that both of these biomolecules could be involved in the onset and progression of PD.

Protein sequencing revealed that amyloid fibrils found in Lewy bodies are formed by α-synuclein (α-Syn), a small protein localized in cell membranes.^4^ Although the exact molecular function of this protein is unclear, it is known that α-Syn regulates neurotransmitter release in synaptic clefts.^5−12^ Specifically, α-Syn binds phospholipids in plasma membranes and synaptobrevin-2 of SNARE complexes.^13−18^ Under pathological conditions, α-Syn can aggregate forming toxic oligomers and fibrils.^19−29^ The rate of protein aggregation could be altered by lipids.^30−32^ Specifically, anionic lipids facilitated protein aggregation, whereas zwitterionic phosphatidylcholine (PC) strongly inhibited α-Syn aggregation.^33^ This effect also depends on the protein-to-lipid (P/L) ratio.^30−32^ Galvagnion and co-workers showed that at low P/L, lipids accelerate α-Syn aggregation, whereas with an increase in P/L, a decrease in the rate of α-Syn aggregation was observed.^30−32^ Our group found that lipids not only altered the rate of α-Syn aggregation but also uniquely changed the secondary structure of α-Syn oligomers and fibrils.^33,34^ As a result, α-Syn aggregates formed in the presence of lipid membranes exerted significantly higher toxicity to rat dopaminergic cells compared to α-Syn oligomers and fibrils formed in the lipid-free environment.^33,34^

Jakubec and co-workers found that in lipid membranes, cholesterol (Cho) enhances protein-membrane interactions.^35^ This results in acceleration of α-Syn aggregation. Similar effects of Cho were observed by Zhaliazka and co-workers on amyloid β_1–42_ (Aβ_1–42_),^36^ the amyloid protein associated with the etiology and progression of Alzheimer’s disease (AD). Specifically, the researchers found that Cho accelerated protein aggregation and modified the secondary structure of Aβ_1–42_ oligomers and fibrils, which, in turn, enhanced the cytotoxicity of these protein aggregates. Specifically, it was found that 5% Cho in PC large unilamellar vesicles (LUVs) increased the rate of Aβ_1–42_ aggregation and enhanced toxicity of oligomers observed 3 and 22 h. These findings indicate that Cho plays a very important role in AD. However, it remains unclear whether this effect is linked to the presence of Cho or changes in the concentration of this physiologically important lipid play the key role in α-Syn aggregation.^37^ To end this, we investigate the effect of LUVs that contain low (15%), normal (30%), and high (60%) concentrations of Cho on α-Syn aggregation. We first exposed *Caenorhabditis elegans* that overexpressed α-Syn to diets with different concentrations of Cho. We found that a high (60%) concentration of Cho in the *C. elegans* diet caused a drastic shortening of their lifespan. In vitro experiments showed that high concentrations of Cho accelerate the rate of α-Syn aggregation and increase the toxicity of α-Syn fibrils. These results suggest that Cho-linked changes in the diet could be the potential cause of the onset and spread of PD.

## Results

### Changes in the Dietary Composition of *C. elegans* Alter Their Lifespan

One can expect that changes in the intake of Cho could alter the rate of α-Syn aggregation and toxicity of amyloid aggregates, based on previous evidence of protein–lipid interactions. To test this hypothesis, we used NL5901 *C. elegans* that endogenously overexpress α-Syn. *C. elegans* were kept on the media supplied with LUVs composed of 60:40, 30:70, and 15:85 Cho and DPPC, as well as 100% DPPC. We also exposed *C. elegans* to the media with no LUVs present (control). Finally, we used wild-type N2 *C. elegans* that did not overexpress α-Syn as a control to demonstrate that changes in the lifespan of NL5901 *C. elegans* were linked to α-Syn overexpression and were not caused by lipids themselves.

**Figure 1:**
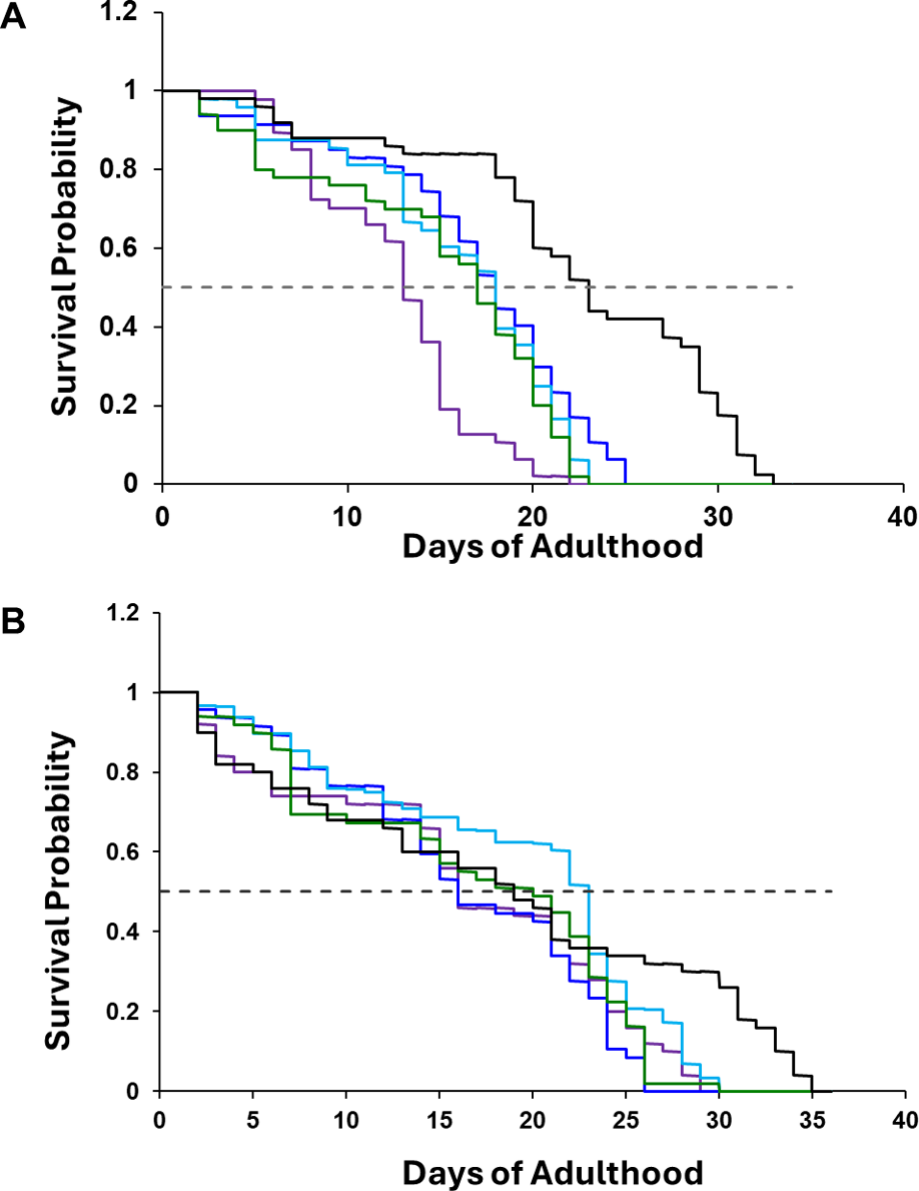
Lipids alter the toxicity of α-Syn aggregates in *C. elegans*. Kaplan–Meier survival probability curves for NL5901 (A) and N2 WT (B) *C. elegans* with dietary lipid supplementation of LUVs with 60:40 Cho/DPPC (purple), 30:70 Cho/DPPC (blue), 15:85 Cho/DPPC (light blue), and DPPC (green), as well as lipid-free environment (black). Dashed line (---) indicates p50 of the survival probability.

We found that the presence of LUVs with 30:70, 15:85 Cho, and DPPC, as well as 100% DPPC drastically reduced the lifespan of NL5901 *C. elegans*, whereas this effect was entirely absent in N2 *C. elegans*, Figure 1. We found that an increase in the intake of Cho (60:40 Cho/DPPC) by NL5901 *C. elegans* caused an even stronger decrease in the lifespan of the worms, Figure 1. These findings indicate that the onset and progression of PD could be linked to an increase in the amount of consumed Cho.

**Figure 2:**
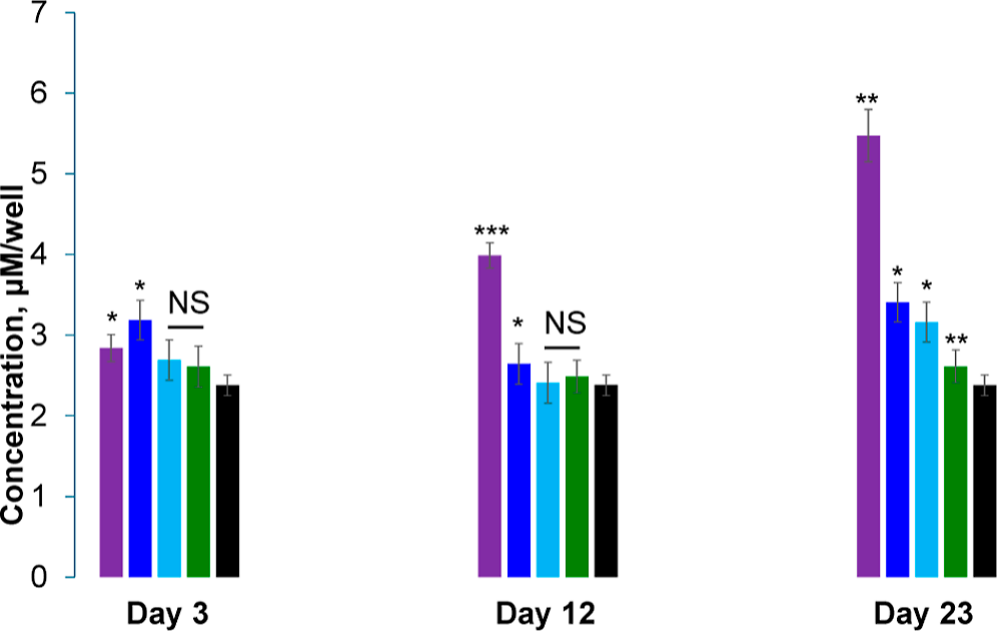
Lipids alter the concentration of α-Syn in NL5901 *C. elegans*. Histograms of ELISA of α-Syn possessed by NL5901 *C. elegans* that were kept on lipid-free diet (black), as well as diet supplied with 60:40 Cho/DPPC (purple), 30:70 Cho/DPPC (blue), 15:85 Cho/DPPC (light blue), and DPPC (green). According to a one-way ANOVA, *P < 0.05, **P < 0.01, ***P < 0.001; NS is nonsignificant difference.

Next, we utilized ELISA to demonstrate that the observed changes in the lifespan were linked to the accumulation of α-Syn in *C. elegans*. We found that *C. elegans* with 60:40 Cho/DPPC and 30:70 Cho/DPPC in their dietary lipid supplementation accumulated significantly higher concentrations of α-Syn already at day 3 compared to *C. elegans* kept on the lipid-free diet, Figure 2. The same difference between *C. elegans* that was kept on 60:40 Cho/DPPC, 30:70 Cho/DPPC, and *C. elegans* kept on the lipid-free diet was observed at day 12. We found that at day 23, *C. elegans* that were kept on 60:40 Cho/DPPC had a significantly greater amount of α-Syn compared to worms exposed to other diets. We also found an increase in the concentration of α-Syn in *C. elegans* that were kept on diets with lipid supplements compared with worms that were exposed to lipid-free media. These results demonstrate that the lifespan of NL5901 *C. elegans* had a direct relationship with the accumulation of α-Syn, which in turn is linked to the presence of lipids in the worm diets.

### In Vitro Analysis of α-Syn Aggregation in the Presence of LUVs Composed of Different Cho/DPPC Ratios

**Figure 3:**
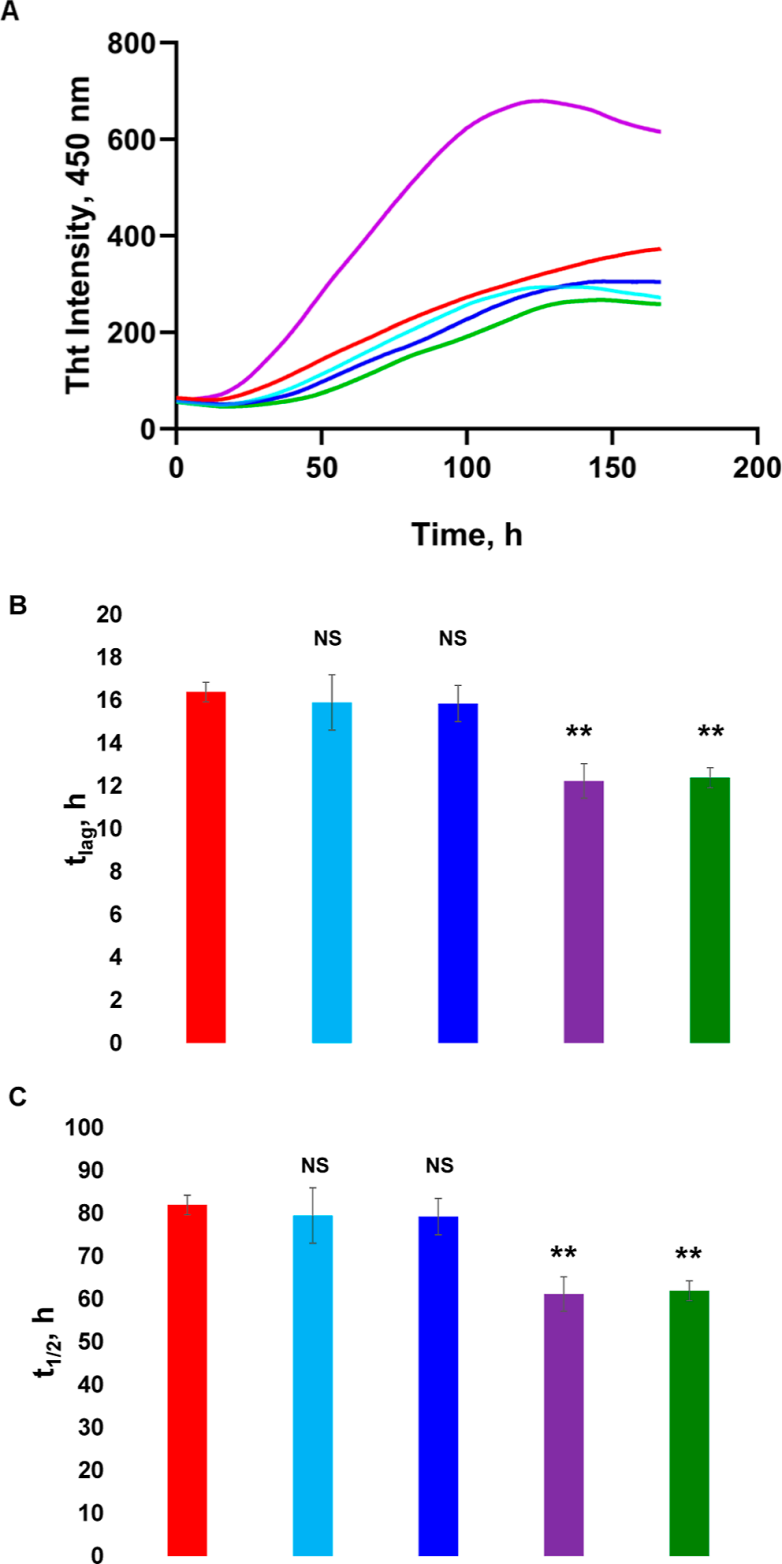
Lipids alter rates of α-Syn aggregation. ThT kinetics (A) with corresponding *t*_lag_ (B) and *t*_1/2_ (C) of α-Syn aggregation in the lipid-free environment (red), as well as in the presence of LUVs that contained 60:40 (purple), 30:70 (blue), 15:85 (light blue) of Cho/DPPC, and 100% DPPC (green). Lag-phase time (*t*_lag_) corresponds to 10% increase in the ThT intensity, whereas half-time (*t*_1/2_) corresponds to 50% of the maximal ThT intensity observed in the kinetic measurements. Excitation 450 nm; emission 488 nm. According to a one-way ANOVA, **P < 0.01, NS is nonsignificant difference.

We used the thioflavin T (ThT) assay to elucidate whether different ratios of Cho in LUVs would alter the rate of α-Syn aggregation. In the lipid-free environment, α-Syn aggregated with a lag-phase (*t*_lag_) of ∼16.4 h that was followed by a rapid increase in ThT fluorescence, Figure 3. This increase indicated the formation of amyloid fibrils. ThT assay revealed similar *t*_lag_ for α-Syn aggregated with LUVs composed of 15:85 (*t*_lag_ = 15.9 h) and 30:70 (*t*_lag_ = 15.8 h) Cho/DPPC. However, we found that LUVs composed of 60:40 Cho/DPPC (*t*_lag_ = 12.2 h) and 100% DPPC (*t*_lag_ = 12.3 h) drastically shortened *t*_lag_ of α-Syn aggregation. These results indicate that high concentrations of Cho in plasma membranes decrease the lag-phase of α-Syn aggregation.

The same conclusions could be made about the *t*_1/2_ of ThT assays that indicate the rate of protein aggregation. We found that LUVs composed of 60:40 Cho/DPPC drastically accelerated the rate of α-Syn aggregation (*t*_1/2_ = 61 h) compared to all lipid-free environment (*t*_1/2_ = 82 h), Figure 3. This effect was not observed for LUVs composed of 15:85 (79.5 h) and 30:70 (79.3 h) Cho/DPPC. These results indicate that high concentrations of Cho in lipid membranes accelerate the rate of α-Syn aggregation. Finally, we observed slightly different intensities of ThT signals in all samples at the late stages of protein aggregation (plateaus > 140 h). Such differences could originate from the different number of protein aggregates present in these samples. Alternatively, these differences in the intensity of ThT signals could be caused by dissimilar surface properties of amyloid fibrils that were induced by lipids present in the samples.

It should be noted that Dou and co-workers previously reported that the presence of LUVs composed of C14:0 PC (DMPC) strongly suppressed α-Syn aggregation.^33^ However, such effects were not observed for DPPC (Figure 3). Therefore, we can conclude that a change in the two carbon atoms in the fatty acids of PC caused a major effect on the aggregation of α-Syn.

### Morphological Examination and Structural Characterization of α-Syn Aggregates Grown in the Presence of Lipids and in the Lipid-Free Environment

**Figure 4:**
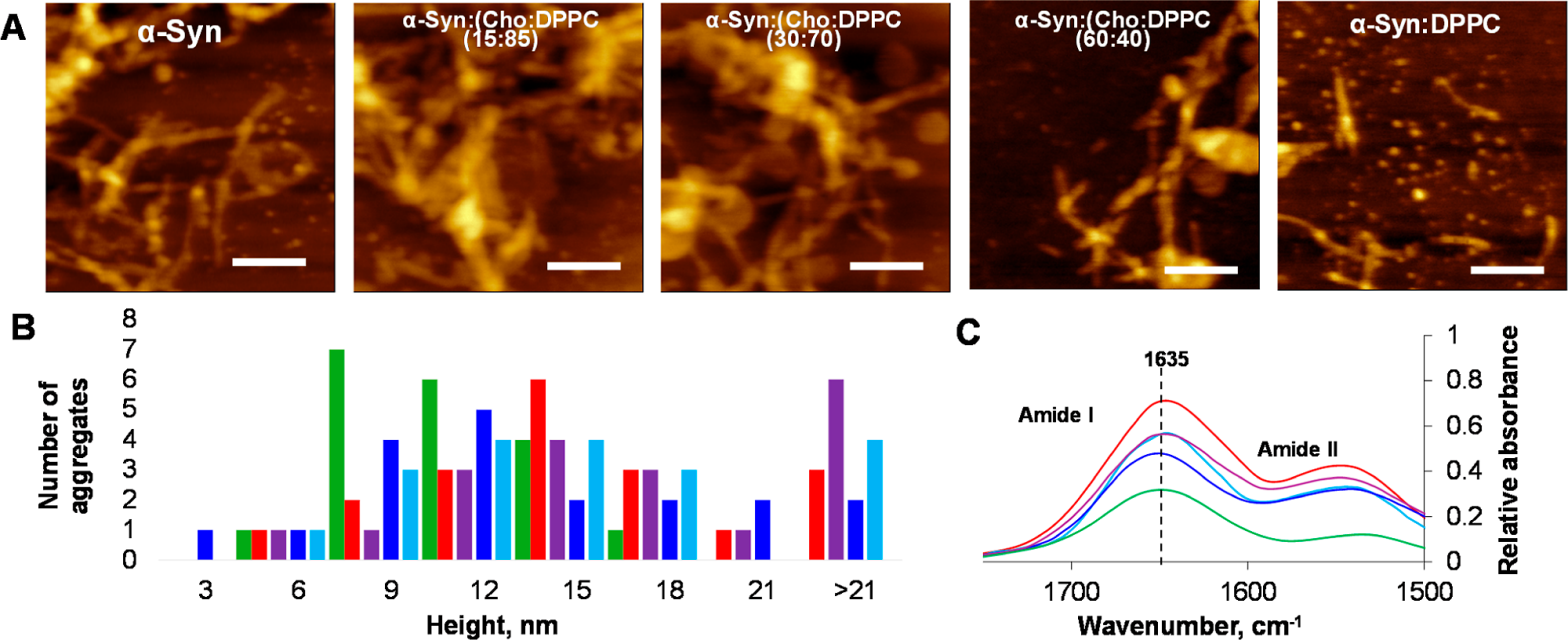
Morphological examination and structural characterization of α-Syn aggregates grown in the presence of lipids and in the lipid-free environment. AFM images (A) of α-Syn aggregates formed in the lipid-free environment, as well as in the presence of LUVs that contained 60:40, 30:70, 15:85 of Cho/DPPC, and 100% DPPC. Scale bars are 500 nm. Histogram (B) of height distribution of protein aggregates observed in α-Syn (red), α-Syn:(Cho/DPPC (15:85)) (light blue), α-Syn:(Cho/DPPC (30:70)) (blue), α-Syn:(Cho/DPPC (60:40)) (purple), and α-Syn:DPPC (green). Same number of protein aggregates was analyzed in each sample. FTIR (C) spectra of α-Syn aggregates grown in the lipid-free environment (red), as well as in the presence of LUVs that contained 60:40 (purple), 30:70 (blue), 15:85 (light blue) of Cho/DPPC, and 100% DPPC (green).

Morphological analysis of α-Syn aggregates grown in the lipid-free environment revealed the presence of long thick fibrils with an average height of 15 nm, as shown in Figure 4. Morphologically similar aggregates were observed in all α-Syn:(Cho/DPPC) samples. Some of these fibrils were assembled into higher order supramolecular clusters. At the same time, oligomers and much thinner fibrils (6–12 nm in height) were observed in α-Syn:DPPC, as shown in Figure 4. These results indicate that the presence of Cho does not alter the morphology of α-Syn fibrils. However, DPPC caused the formation of only thin filaments and oligomers that were not observed in other samples. IR spectroscopy was utilized to determine the secondary structure of α-Syn aggregates grown in the presence of lipids and in the lipid-free environment. We found that IR spectra acquired from all samples exhibit amide I and II bands, Figure 4C. The position of the amide I band can be used to interpret the secondary structure of proteins. We found that amide I in all acquired spectra was centered around 1635 cm^–1^, which indicates the predominance of parallel β-sheet in α-Syn fibrils formed in the presence and absence of lipids.

### Cytotoxicity of α-Syn Aggregates Grown in the Presence of Lipids and in the Lipid-free Environment

**Figure 5:**
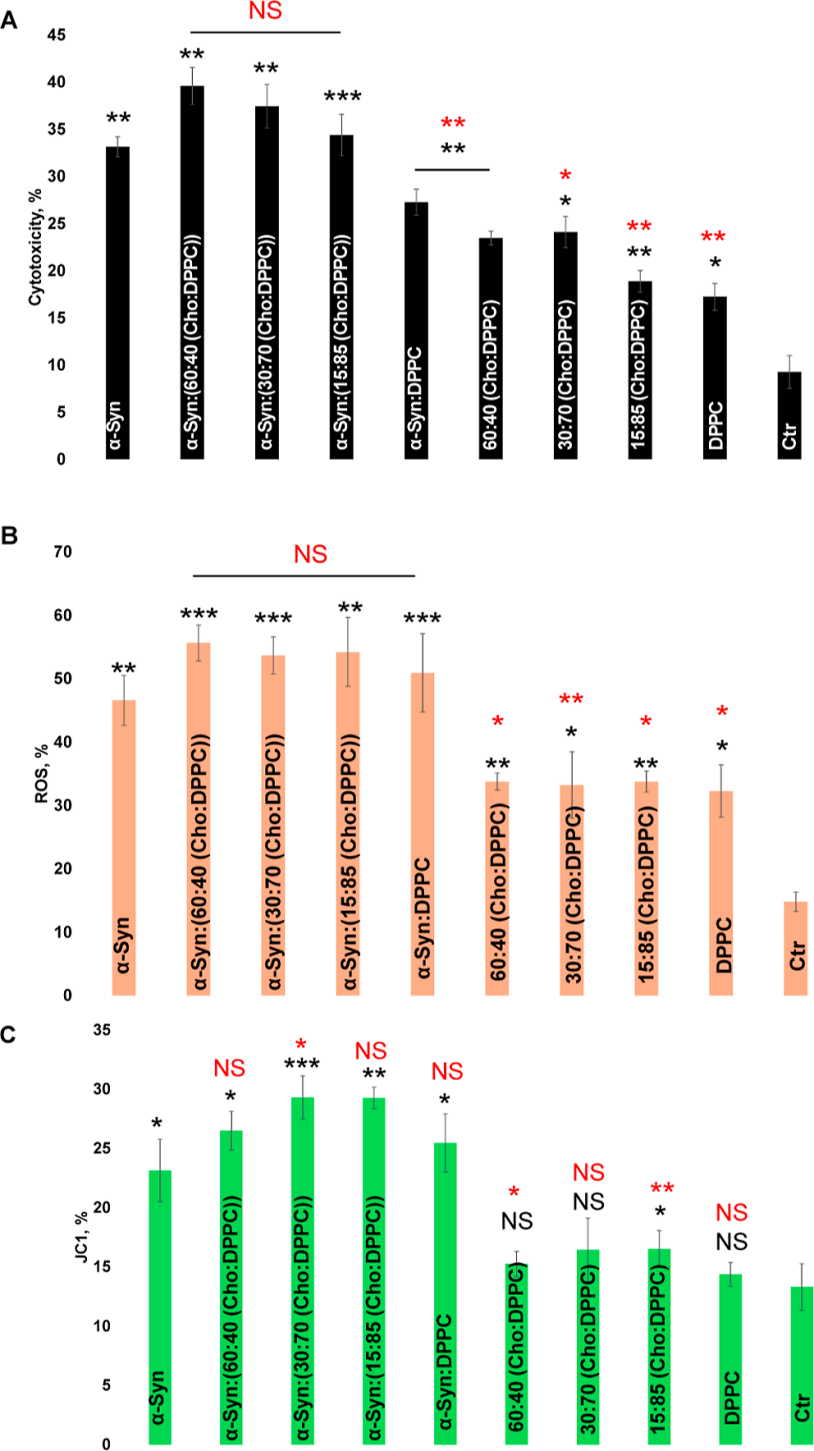
Histograms of LDH (A), ROS (B), and JC-1 (C) toxicity assays of α-Syn aggregation in the lipid-free environment (red), as well as in the presence of LUVs that contained 60:40, 30:70, and 15:85 of Cho/DPPC, as well as 100% DPPC (green). Black asterisks (*) show a significant level of differences between α-Syn and α-Syn aggregates grown in the presence of lipids, as well as between lipid samples and the control. Red asterisks (*) show a significant level of differences between α-Syn aggregates grown in the presence of and absence of lipids. According to a one-way ANOVA, *P < 0.05, **P < 0.01, ***P < 0.001, NS is nonsignificant difference.

We utilized rat dopaminergic neuronal cells to examine the cytotoxicity of α-Syn aggregates assembled in the presence of LUVs with different concentrations of Cho. LDH assay showed that α-Syn fibrils formed in the presence of different concentrations of Cho exerted similar cytotoxicity to α-Syn fibrils assembled in the lipid-free environment, Figure 5. At the same time, α-Syn:DPPC fibrils were significantly less toxic compared with α-Syn fibrils. Based on these results, we can conclude that the presence of Cho does not change, while DPPC lowers the cytotoxicity of α-Syn fibrils. Similar results were obtained by the ROS assay. Specifically, we found that α-Syn fibrils formed in the presence of different lipids triggered a very similar ROS response in N27 rat dopaminergic cells. We found that lipids themselves also caused increased levels of ROS compared to the control.

We also used the JC1 assay to examine the extent to which α-Syn aggregates formed in the presence of LUVs with different concentrations of Cho damage cell mitochondria. Our results showed that α-Syn fibrils formed in the presence of different lipids caused a similar magnitude of mitochondrial impairment, Figure 5.

It was previously demonstrated by our^33,34,38^ and other research groups^10,39,40^ that α-Syn interacts with lipids via electrostatic and hydrophobic interactions, making protein–lipid complexes. Such complexes aggregate, forming morphologically and structurally different protein aggregates compared to the amyloid fibrils assembled in the lipid-free environment. We also demonstrated that α-Syn aggregates formed in the presence of lipids exerted significantly higher cytotoxicity compared with fibrils assembled in the absence of lipids. The same conclusions could be made about the rate of α-Syn aggregation in the presence of lipid vesicles. Our current results indicate that Cho significantly accelerated α-Syn aggregation. We infer that this effect could be caused by an increase in the hydrophobicity of LUVs that contain Cho compared to that of DPPC LUVs. The same effect of Cho could be expected in *C. elegans* that exhibit shorter lifespan if exposed to diets with high concentrations of Cho.

Our results also indicate that the presence of Cho does not alter the toxicity effects exerted by α-Syn fibrils to neurons. Additional studies are required to examine mechanisms of α-Syn fibril toxicity to reveal whether the observed morphological differences in α-Syn fibrils caused by the presence of Cho could alter the extent to which amyloid aggregates are endocytosed by the cells. This information will shed light on possible damage caused by amyloids to the cell endoplasmic reticulum and other important organelles.

### Conclusions

Our results showed that a high concentration of Cho in lipid membranes accelerated the rate of α-Syn aggregation. Using *C. elegans* as a life model, we demonstrated that an increase in the rate of α-Syn aggregation results in the accumulation of greater amounts of α-Syn aggregates, which causes a decrease in *C. elegans* lifespan. We also found that α-Syn formed at different concentrations of Cho had slightly different morphologies. However, these aggregates exert similar levels of toxicity to N27 rat dopaminergic neurons compared with α-Syn fibrils formed in the lipid-free environment. Our results also demonstrate a direct relationship between the lipid supplement in the diet and the rate of α-Syn aggregation. Based on these results, one can expect that diet-linked changes in the concentration of Cho in plasma membranes could be the underlying cause of PD.

## Methods

### Materials

DPPC and Cho were purchased from Avanti (Alabaster, AL, USA).

### Protein Expression and Purification α-Syn

*Escherichia coli* BL21 (DE3), strain Rosetta was transformed with pET21a-α-Syn plasmid. *E. coli* cultures were grown in the media to reach an appropriate cell density. Next, 1 mM IPTG was added to the medium to induce α-Syn expression. After that, the bacterial culture was centrifuged at 8000 rpm for 10 min. The pellet was harvested and resuspended in lysis-Tris buffer (10 mM EDT, 50 mM Tris, and 150 mM NaCl, pH 7.5). The suspended cells were exposed to 78 °C for 30 min to lyse *E. coli* cells. Next, samples containing α-Syn were centrifuged at 16,000*g* for 40 min. The supernatant containing α-Syn was collected. To precipitate bacterial proteins and contaminants, streptomycin sulfate (10% solution, 136 μL/mL) in glacial acetic acid (228 μL/mL) was added to the supernatant. The mixture was then centrifuged at 16,000*g* for 10 min at 4 °C. Afterward, the supernatant was precipitated by saturated ammonium sulfate ((NH_4_)_2_SO_4_) at 4 °C, and samples were centrifuged to collect an α-Syn pellet that was washed one more time with (NH_4_)_2_SO_4_ at 4 °C (a 1:1 v/v mixture of saturated (NH_4_)_2_SO_4_ and water). Finally, the pellet was resuspended in 100 mM ammonium acetate (NH_4_(CH_3_COO)) under constant stirring for 5 min. Next, absolute ethanol was added to the resuspended α-Syn. The mixture was then subjected to ethanol precipitation. This step was repeated twice at room temperature. Finally, collected pellets were dissolved in phosphate buffered saline (PBS).

### Size Exclusion Chromatography (SEC)

Samples were first centrifuged at 14,000*g* for 30 min using a benchtop microcentrifuge (Eppendorf centrifuge 5424 USA). Next, 500 μL of the concentrated α-syn was injected in an AKTA pure (GE Healthcare) FPLC system equipped with a gel filtration column (Superdex 200 10/300). Samples were eluted isocratically using PBS, pH 7.4, at a flow rate of 0.5 mL/min. All protein purification was done at 4 °C. During the FPLC run, 1.5 mL fractions were collected based on UV–vis detection at 280 nm.

### Liposome Preparation

LUVs of Cho/DPPC were prepared according to the method proposed by Galvagnion et al.^32^ Briefly, Cho and PC at 60:40, 30:70, and 15:85 mol/mol ratios were mixed in chloroform. Once all the solvent was dried, the lipid mixture was dissolved in PBS, pH 7.4. Next, the lipid solution was heated in a water bath to ∼50 °C for 30 min and then immersed into liquid nitrogen for 3–5 min. This procedure was repeated 10 times. Finally, the lipid solution was processed using an extruder equipped with a 100 nm membrane (Avanti, Alabaster, AL, USA). Dynamic light scattering was used to ensure that the size of LUVs was within 100 ± 10 nm.

### Protein Aggregation

α-Syn was dissolved in PBS to reach the final protein concentration of 40 μM. Protein concentration was determined using a NanoDrop. In parallel, α-Syn and LUVs were mixed at a 1:1 molar ratio in PBS. Samples were incubated in a 96 well-plate at 37 °C for 160 h under 510 rpm agitation in the plate reader (Tecan, Männedorf, Switzerland)

### Kinetic Measurements

Rates of α-Syn aggregation were measured using a ThT fluorescence assay. For this, samples were mixed with 2 mM ThT solution and placed in a 96 well-plate that was kept in the plate reader (Tecan, Männedorf, Switzerland) at 37 °C for 160 h under 510 rpm agitation. Fluorescence measurements were taken every 10 min (excitation 450 nm; emission 488 nm).

### AFM Imaging

Microscopic imaging of α-Syn aggregates was performed on AIST-NT-HORIBA system (Edison, NJ) using silicon AFM probes (force constant 2.7 N/m; resonance frequency 50–80 kHz) purchased from AppNano (Mountain View, CA, USA). Preprocessing of the collected AFM images was made using AIST-NT software (Edison, NJ, USA).

### Attenuated Total Reflectance Fourier-Transform Infrared (ATR-FTIR) Spectroscopy

An aliquot of the protein sample was placed onto the ATR crystal and dried at room temperature. Spectra were measured using a Spectrum 100 FTIR spectrometer (Perkin-Elmer, Waltham, MA, USA). For each measurement, 3 spectra were collected and averaged using Thermo Grams Suite software (Thermo Fisher Scientific, Waltham, MA, USA).

### Cell Culturing

Rat midbrain dopaminergic neuronal N27 cells were purchased from ATCC and grown in RPMI 1640 Medium (Thermo Fisher Scientific, Waltham, MA, USA) with 10% fetal bovine serum (FBS) (Invitrogen, Waltham, MA, USA). For experimentation, cells were seeded in a 96 well-plate (5000 cells per well) at 37 °C under 5% CO_2_. After several hours, cells were found to fully adhere to the wells, reaching ∼70% confluency.

### Cell Toxicity Assay

After 24 h of incubation, a lactate dehydrogenase assay was performed on the cell medium using the CytoTox 96 nonradioactive cytotoxicity assay (G1781, Promega, Madison, WI, USA). Absorption measurements were made in a plate reader (Tecan, Mannedorf, Switzerland) at 490 nm. Every well was measured 25 times in different locations.

ROS and JC-1 Assays were performed as previously described.^41^ Rat N27 neurons were cultured in 96-well plates in RPMI 1640 with 10% FBS. After cells reached 80% confluency, media was replaced with DMEM that contained 2.5% FBS and amyloid aggregates. Controls were treated with an equal volume of PBS or an equal concentration of lipids. ROS and JC-1 assays were performed 24 h after treatment. All conditions were performed in triplicates.

### *C. elegans*

*C. elegans* strain NL5901 was purchased from the University of Minnesota worm bank, and N2 wildtype worms were a kind gift from Dr. Michael Polymenis of Texas A&M University. Worms were maintained at 20 °C on NGM plates seeded with OP50 *E. coli* and allowed to reach an egg-producing age before age synchronizing as previously described.^42^ Synchronized worms were allowed to reach day 1 adult age before 10 worms were moved onto each experimental plate. All experimental plates were made as previously described.^42^ Lipid supplementation was performed by mixing concentrated stocks with 10× concentrated OP50 before seeding, quickly drying, and UV irradiating. Counts of alive and dead worms were taken daily until all of the worms had died. Survival probability was calculated by using the Kaplan–Meier survival curve equation.
